# Long-term psychosocial outcome following mild traumatic brain injury and minor stroke: a direct longitudinal comparison

**DOI:** 10.1007/s00415-020-10385-6

**Published:** 2021-01-13

**Authors:** Daan P. J. Verberne, Rudolf W. H. M. Ponds, Mariëlle E. A. L. Kroese, Melloney L. M. Wijenberg, Dennis G. Barten, Raphaël Pasmans, Julie Staals, Caroline M. van Heugten

**Affiliations:** 1 Faculty of Health, Medicine and Life Sciences, School for Mental Health and Neuroscience (MHeNs), Maastricht University Medical Center, Maastricht University, P.O. 616 UNS 40, 6200 MD Maastricht, The Netherlands; 2 Limburg Center for Brain Injury, Maastricht, The Netherlands; 3 Department of Brain Injury Rehabilitation, Adelante Rehabilitation Centre of Expertise in Rehabilitation and Audiology, Hoensbroek, The Netherlands; 4 Department of Medical Psychology, Maastricht University Medical Centre, Maastricht, The Netherlands; 5 Department of Health Services Research, Faculty of Health, Medicine and Life Sciences, Maastricht University, Maastricht, The Netherlands; 6 Department of Neuropsychology and Psychopharmacology, Faculty of Psychology and Neuroscience, Maastricht University, Maastricht, The Netherlands; 7 Department of Emergency Medicine, VieCuri Medical Center, Venlo, The Netherlands; 8 Department of Neurology, Zuyderland Medical Center, Heerlen, The Netherlands; 9 Department of Neurology, Maastricht University Medical Center, Maastricht, The Netherlands

**Keywords:** Stroke, Brain injuries, traumatic, Longitudinal studies, Social participation, Emotional adjustment, Primary health care

## Abstract

**Background and purpose:**

Research suggests comparable long-term psychosocial outcomes following mild traumatic brain injury (mTBI) and minor stroke, but no direct comparison has been made. This study aimed to directly compare psychosocial outcome over time in persons with mTBI and minor stroke.

**Methods:**

In this multicenter, prospective longitudinal cohort study, community-dwelling persons with mTBI (*n* = 182) and minor stroke (*n* = 48) were assessed at 6 weeks, 3, 6 and 12 months post-injury. Outcome measures included anxiety and depression symptoms (Hospital Anxiety and Depression Scale—HADS), cognitive problems in daily life (Checklist for Cognitive and Emotional Consequences of Stroke—CLCE-24) and quality of life (EuroQol-5D-5L—EQ-5D-5L). Multilevel growth curve modeling, controlled for demographic variables, was used to determine outcomes over time between groups. Proportions of persons reporting persistent psychosocial symptoms at 6 months post-injury were compared using Pearson’s Chi-squared tests.

**Results:**

Improvements in outcomes were observed in the first 6 months and effects stabilized to 12 months post-injury in both groups. Minor stroke cases reported significantly higher levels of HADS anxiety and a significantly reduced increase in EQ-5D-5L utility scores than mTBI cases, but differences were small in absolute numbers. No significant differences were observed between groups regarding HADS depression and CLCE-24 cognition scores. Proportions of persons reporting persistent psychosocial symptoms were equal between groups.

**Conclusions:**

Psychosocial outcome is largely comparable following mTBI and minor stroke. Specific attention should be paid to anxiety symptoms and cognitive problems in daily life for which uniform aftercare seems appropriate.

## Introduction

Brain injuries are among the most disabling chronic conditions worldwide [1–3], of which the most frequently occurring are of a traumatic and cerebrovascular nature (stroke) [2, 3]. Numbers of both conditions keep increasing worldwide because of the aging population [2, 3]. Overall, the majority of traumatic brain injuries (TBI) (80–90%) [1] and strokes (57%) [4] are of mild severity. Although we acknowledge the differences in pathophysiological processes underlying the forms of brain injury, in the longer term the two groups become more similar regarding deficits and course of treatment. Persons with mild brain injury are usually community residing, having minor motor and communication deficits, being discharged home without rehabilitation or aftercare because of expected full functional recovery [5, 6]. However, it is estimated that 20–25% of these community-residing, ‘walking and talking’ persons with mild brain injury do not recover and report persisting psychosocial symptoms including mostly emotional and cognitive problems [6–9].

Recovery following brain injury stabilizes typically after 6 months which can cause symptoms to persist into the long term if left unattended [6, 10, 11]. Anxiety and depressive symptoms persist in 23 and 16% of mild TBI cases (mTBI), respectively [12]. It is estimated from stroke registries that 29% of community-dwelling persons report long-term anxiety symptoms and 24% report depressive symptoms [13]. Estimates of cognitive problems vary depending on assessment methods, but persist into the long term in around 50% of mTBI cases [14] and approximately 35% of minor stroke cases [9]. Quality of life (QoL) is negatively affected by these persisting symptoms, because of confrontation with difficulties on a daily basis and trouble managing daily activities [15]. Despite these long-term consequences, coordinated attention for psychosocial symptoms in aftercare is currently missing for both (m)TBI and (minor) strokes.

Research suggests that long-term psychosocial outcomes following mTBI and minor stroke are comparable, but a direct and longitudinal comparison between injuries has not been performed to the best of our knowledge. Studies have included either mixed samples of brain injuries [16–18], mixed severities [19] or made a direct comparison cross-sectionally [20]. A direct longitudinal comparison could support the seemingly comparable psychosocial outcome over time between injuries which would justify a transdiagnostic design of uniform aftercare.

This study primarily aimed to enable a direct longitudinal comparison of emotional functioning, cognitive problems in daily life, and QoL in the first year after mTBI and minor stroke. Secondary, we compared proportions of persons experiencing persistent symptoms at 6 months between groups. We hypothesized comparable long-term psychosocial outcome between groups, regarding recovery over time and persistent psychosocial symptomatology at 6 months.

## Methods

### Design

The current study is part of the COmplaints, HEalthcaRE Needs and cosTs (COHERENT) cohort study which is a multicenter, prospective, longitudinal observational study following persons with mild to moderate acquired brain injury from onset to 1 year post-injury. The aim of the COHERENT study was to obtain a comprehensive overview and investigate the psychosocial well-being, healthcare consumption and healthcare needs of community-dwelling persons over the course of 1 year following brain injury.

### Participants

Persons with mild or moderate brain injury were included in the COHERENT cohort study between April 2017 and October 2018 by five participating hospitals in the Southern part of the Netherlands. Persons eligible for participation were adults (> 18 years of age) who suffered from TBI, stroke or other forms of acquired brain injury of mild to moderate severity as diagnosed by a clinician, within the past 6 weeks and were discharged home after visiting the emergency department (ED) or hospital admission. Persons were excluded if they were legally incompetent, insufficiently capable to follow up for 1 year or had insufficient command of the Dutch language to understand and complete the questionnaires, as judged by the clinician. Severity of TBI and stroke were assessed with the Glasgow Coma Scale (GCS) and the National Institutes of Health Stroke Scale (NIHSS) score at admission, respectively. The current study selected persons with mTBI and minor stroke from the COHERENT cohort. Identification of mTBI was performed according to the clinical criteria of the World Health Organization (WHO) Collaborating Center Task Force. These criteria include a GCS of 13–15 and one of the following symptoms: confusion or disorientation, post-traumatic amnesia (< 24 h), loss of consciousness (< 30 min) or other transient neurological symptoms such as focal signs, seizures or intracranial lesions not requiring surgery [21]. Cases not meeting the criteria of mTBI, (GCS score of 15 and no additional symptoms) were not selected for this study and considered head injuries. Minor stroke was defined according to a NIHSS score of 1–4 [22].

### Procedure

Eligible persons were informed of the study by the treating physician at the emergency department or neurology outpatient clinic. If interested, they received an information leaflet and their contact details were sent to the researcher who provided more study information by telephone. If willing to participate, written study information, an informed consent form and the first questionnaire were sent to the person’s home. This first assessment took place within the first 6 weeks after brain injury (T0). Follow-up assessments took place at 3 (T1), 6 (T2) and 12 months post-injury (T3). If the signed informed consent form and the first questionnaire were not returned by the 6-week deadline, they could not participate in follow-up assessments.

### Measures

#### Demographic and medical information

Demographic information was collected from T0 questionnaires and included age, sex, educational level, pre-injury working status, relationship status, and living situation. Injury-related information was collected from medical files upon receiving signed informed consent. TBI-related information concerned cause of injury, loss of consciousness (in minutes), post-traumatic amnesia (PTA; in hours), other transient neurological symptoms and substance use at time of injury. TBI severity was assessed with the GCS in which lower scores indicate more severe TBI (range 3–15). Stroke-related information concerned stroke type, hemisphere, location, severity and acute treatment (such as intravenous thrombolysis; yes/no). Stroke severity was assessed with the NIHSS in which higher scores are indicative of more severe stroke symptoms (range 0–30) [22]. Neurological history was collected for both injuries.

#### Anxiety and depression

The Hospital Anxiety and Depression Scale (HADS) consists of 14 items in which higher scores indicate more severe symptoms, for either subscale anxiety or depression (range 0–21) [23]. A score of ≥ 8 is indicative of clinically relevant symptoms on the given domain [24].

#### Cognitive problems

The cognition domain of the Checklist for Cognitive and Emotional Consequences of Stroke (CLCE-24) consists of 13 items scored by absence or presence of symptoms. Higher scores indicate more cognitive problems experienced in daily life (range 0–13) [25]. The CLCE-24 was adapted to the study population by replacing ‘stroke’ with ‘brain injury.’

#### Quality of life

Quality of life was measured by the EuroQol-5D-5L (EQ-5D-5L) which assesses five domains: mobility, self-care, usual activities, pain/discomfort and anxiety/depression. The domains are scored on five levels, from ‘no problems’ to ‘extreme problems’ [26]. Dutch tariffs were used to calculate utility scores which range from − 0.446 (worst health state) to 1 (full health) [27].

### Analyses

The mTBI and minor stroke groups were compared on demographic information using independent sample *t* tests and Pearson Chi-square tests. Injury-related information was described per group.

The course of psychosocial outcomes in the first-year post-injury were modeled by multilevel growth curve analyses with the HADS anxiety and depression, CLCE-24 cognition and EQ-5D-5L utility scores as dependent variables in separate models. All available data could be used with this statistical technique. As a first step, an unconditional means model (without any predictors) was fitted, with random intercepts across persons to account for the fact that repeated measures are correlated within individuals. Second, covariates were added to the model as fixed effects: sex, age, educational level, neurological history, along with the linear function of time as a continuous variable (6 weeks, 3, 6 and 12 months). These were added regardless of improved fit. Third, the quadratic function of time was added if overall fit improved. Fourth, group (type of injury: mTBI/minor stroke) was added as a main effect, along with time × group effects (group × linear time and group × quadratic time) to describe possible distinct courses of complaints over time per injury type. Likelihood ratio tests were used to assess model fit. Random slopes were allowed if the model was significantly better compared to a model with only random intercepts based on likelihood ratio testing. Likewise, covariance structures were specified according to the best fit.

Outcomes at T2 were dichotomized to compare proportions of persons experiencing persistent psychosocial symptoms as recovery after 6 months typically stabilizes. Persistent mood symptoms were defined by individual HADS anxiety and depression score of ≥ 8. The CLCE-24 cognition scores were dichotomized with a cutoff ≥ 2 based on mean of 1.9 (standard deviation = 1.9) in healthy controls [28], and the five most frequently CLCE-24 items scored as present were described. Persistent problems regarding QoL were described by the EQ-5D-5L in case problems were reported on ≥ 2 of the five domains. Pearson Chi-square tests were used to statistically compare the proportions of persons reporting persisting symptoms on the HADS, CLCE-24 and EQ-5D-5L between injury groups.

An alpha of 5% (two-sided) was set for significance testing. All statistical analyses were performed with IBM SPSS Statistics for Macintosh, Version 25.0 (New York).

## Results

A total of 316 persons were included in the COHERENT cohort. Seventy cases of head injury (not meeting criteria of mTBI) (22%) were excluded from analyses, as well as seven cases with moderate brain injuries (2%), two cases of other brain injuries (1%) and seven cases who did not complete baseline measurements (2%). A total of 230 cases were included for analyses of whom 182 (79%) suffered from mTBI and 48 (21%) from minor stroke. Twenty-two cases did not complete all follow-up assessments (lost to follow-up: nine at T1, ten at T2 and three at T3). Demographic and injury-related information is displayed in Table 1. Significant differences were observed between mTBI and minor stroke on the variables sex, age at brain injury, hospital admission rates, neurological history and psychological consultation at any point during the study period (*p* < 0.05).

**Table 1:** Demographic and injury-related characteristics of the samples with mild traumatic brain injury and minor stroke

	mTBI (*n* = 182)		Minor stroke (*n* = 48)	
	*n*	*n* (%), mean (SD) or median (IQR)	*n*	*n* (%), mean (SD) or median (IQR)
Sex (men)	182	91 (50%)	48	36 (75%)*
Age at brain injury (years)	182	56.0 (18.7)	48	66.7 (9.5)*
Educational level (high)	182	52 (29%)	48	11 (23%)
In relationship (yes)	181	123 (68%)	48	39 (81%)
Pre-injury working status (working)	182	82 (45%)	48	15 (31%)
Living situation (with others)	181	142 (78%)	48	40 (83%)
Hospital admission (yes)	181	44 (24%)	48	42 (88%)*
Multidisciplinary outpatient rehabilitation (yes)	182	2 (1%)	51	0 (0%)
Neurological history (yes)	182	37 (20%)	51	19 (40%)*
Psychological consultation^a^ (yes)	182	20 (11%)	51	15 (31%)*
mTBI				
Cause of injury	180			
Fall		87 (48%)		
Traffic		70 (39%)		
Violence		4 (2%)		
Sports		10 (6%)		
Hit by object		7 (4%)		
Other		2 (1%)		
Severity (GCS)^a^	182	15.0 (15–15)		
Loss of consciousness (yes)	173	109 (63%)		
Duration in minutes	91	4.8 (6.1)		
PTA (yes)	181	108 (59%)		
Duration in hours	98	2.2 (4.4)		
Other transient neurological symptoms (yes)	182	83 (46%)		
CT abnormality (yes)	181	29 (16%)		
Substance use at brain injury (yes)	182	38 (21%)		
Minor stroke				
Severity (NIHSS)^a^			48	2.0 (1–3)
Type of stroke (ischemic)			48	46 (96%)
Hemisphere			47	
Left				18 (38%)
Right				18 (38%)
Other				11 (24%)
Location (territory of middle cerebral artery)			46	33 (69%)
Intravenous thrombolysis (yes)			48	8 (17%)

Medians (IQR) are displayed for the GCS and NIHSS, being more informative than means (SD).

*GCS* Glasgow Coma Scale, *IQR* interquartile range, *NIHSS* National Institutes of Health Stroke Scale, *PTA* post-traumatic amnesia, *mTBI* mild traumatic brain injury

*p < 0.05

^a^At any point during the study period

**Fig. 1:**
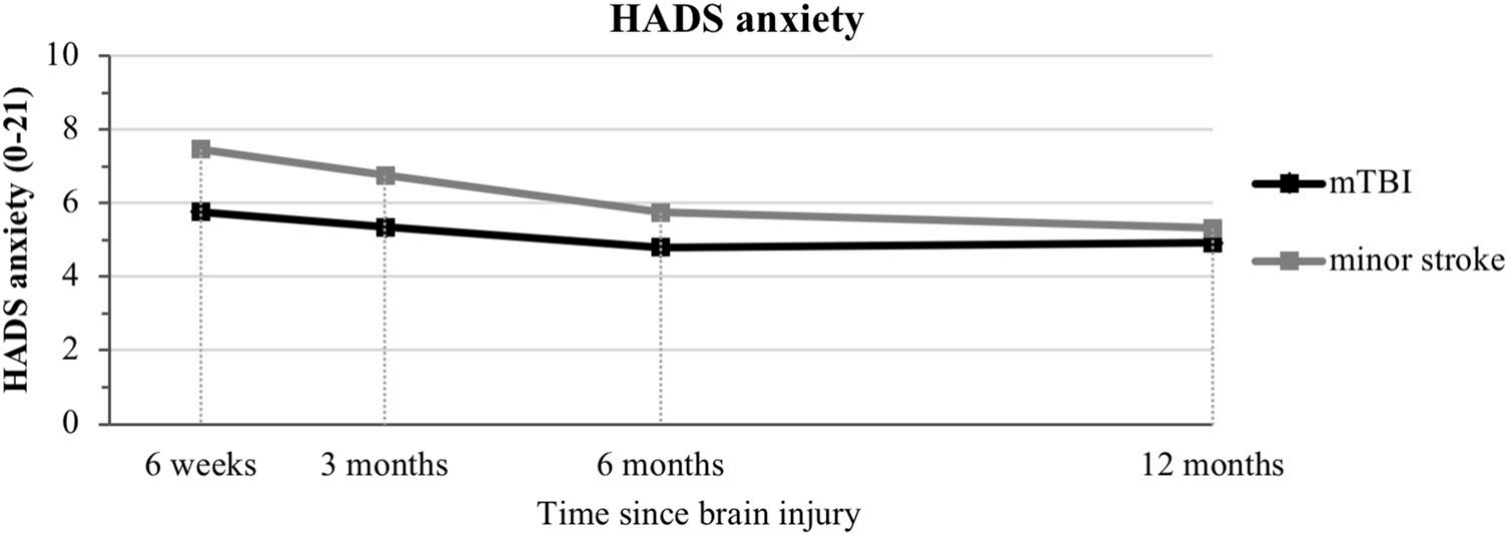
Hospital Anxiety and Depression Scale (HADS) anxiety score displayed over time through multilevel growth curve model estimates

Table 2 shows the results of the multilevel growth models of outcomes over time. Regarding HADS anxiety, no significant interaction (time × group) effect, but a significant main effect was observed, indicating a significant higher HADS anxiety score over time in minor stroke compared to mTBI. Overall, HADS anxiety showed a significant negative linear time effect and a positive quadratic time effect, indicative of a decrease in severity of anxiety symptoms which levels off over time (Fig. 1).

**Table 2:** Multilevel growth modeling of outcomes over time and interaction effects with injury type

	Time effects						Group effect			Interaction effects					
	Linear			Quadratic			Main (stroke = 1)			Linear × group			Quadratic × group		
	Estimate	SE	95% CI	Estimate	SE	95% CI	Estimate	SE	95% CI	Estimate	SE	95% CI	Estimate	SE	95% CI
HADS anxiety	− 0.31	0.11	− **0.52 to **− **0.11**	0.02	0.01	**0.01 to 0.04**	10.70	0.77	**0.18 to 30.21**	− 0.20	0.23	− 0.64 to 0.25	0.01	0.02	− 0.02 to 0.04
HADS depression	− 0.37	0.11	− **59 to **− **0.15**	0.03	0.01	**0.01 to 0.04**	0.10	0.81	− 10.51 to 10.70	0.20	0.24	− 0.28 to 0.68	− 0.02	0.02	− 0.05 to 0.02
CLCE-24 cognition	− 0.30	0.10	− **0.49 to **− **0.10**	0.02	0.01	**0.01 to 0.03**	0.12	0.69	− 10.24 to 10.48	0.20	0.21	− 0.22 to 0.62	− 0.01	0.02	− 0.04 to 0.01
EQ5D5L	0.04	0.01	**0.03 to 0.06**	− 0.003	0.0004	− **0.003 to **− **0.002**	0.06	0.04	− 0.01 to 0.13	− 0.03	0.01	− **0.05 to **− **0.007**	0.002	0.001	**0.0002 to 0.003**

Model covariates: age, sex, level of education, relationship status and neurological history. Significant estimates are displayed by 95% CI in bold (zero not included)

*CI* confidence interval, *CLCE-24* Checklist for Cognitive and Emotional Consequences of Stroke, *EQ-5D-5L* EuroQol 5 domains 5 levels, *HADS* Hospital Anxiety and Depression Scale, *SE* standard error

**Fig. 2:**
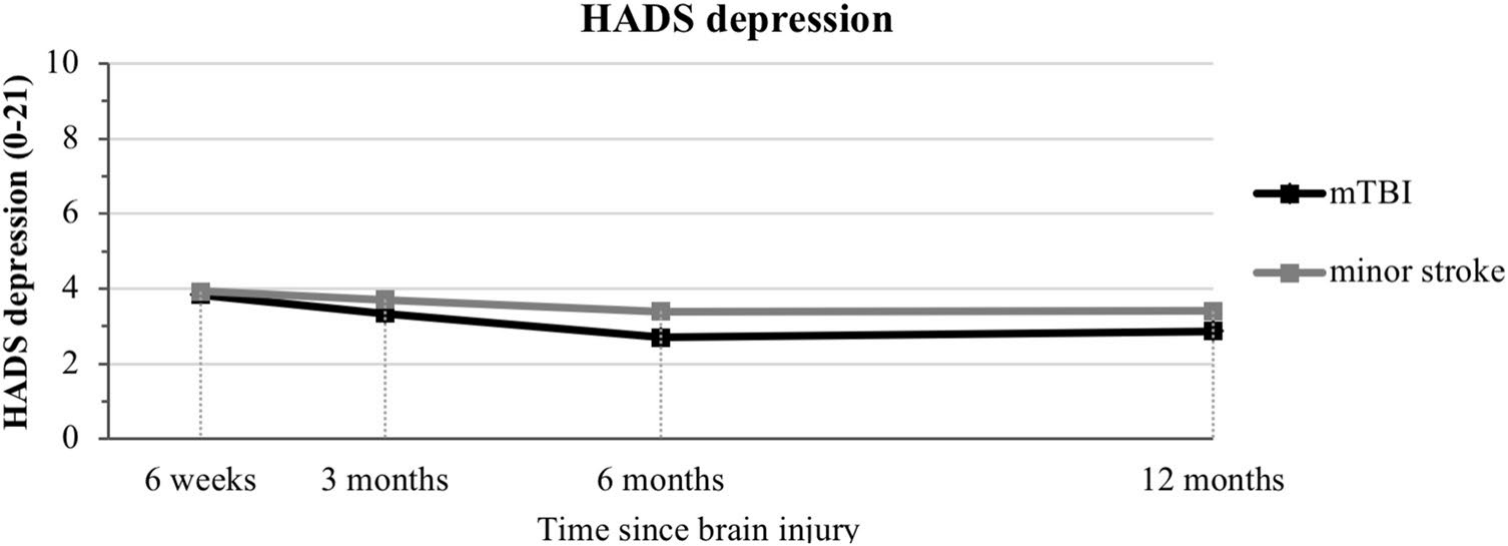
Hospital Anxiety and Depression Scale (HADS) depression score displayed over time through multilevel growth curve model estimates

HADS depression showed no significant (time × group) interaction or main effects, indicative of no significant differences between mTBI and minor stroke over time. Overall, HADS depression showed a significant negative linear time effect and a positive quadratic time effect, indicative of a decrease in severity of depressive symptoms which levels off over time (Fig. 2).

**Fig. 3:**
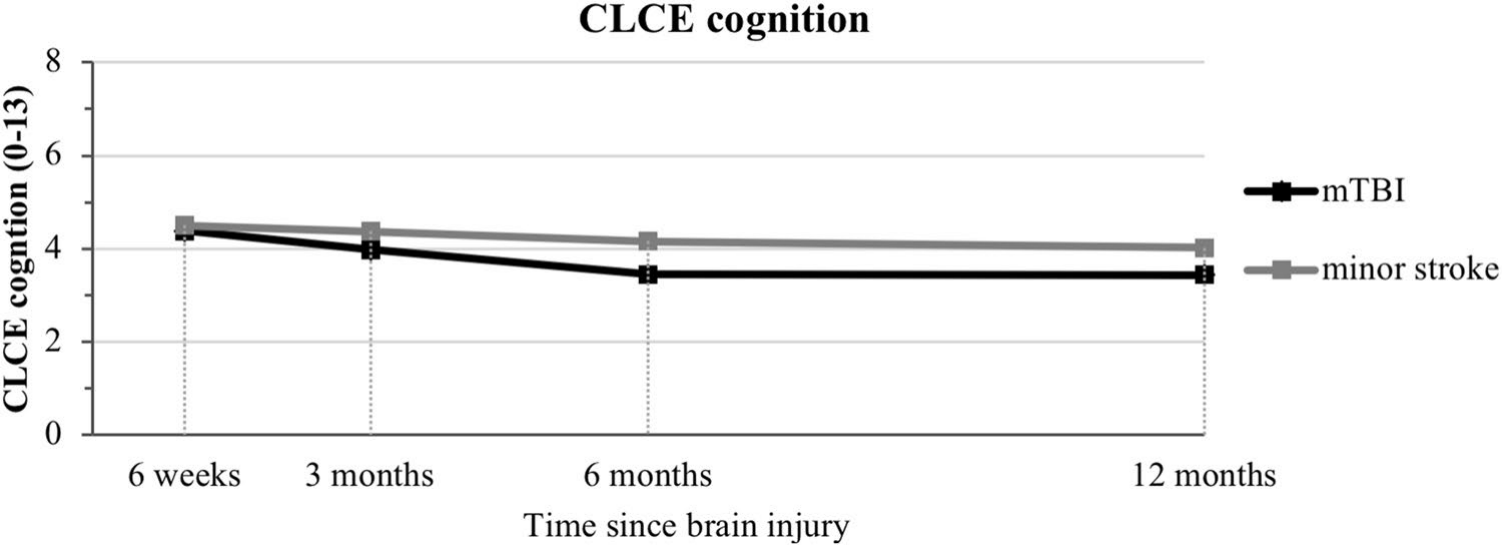
Checklist for cognitive and emotional consequences following stroke (CLCE-24) cognition score displayed over time through multilevel growth curve model estimates

CLCE-24 cognition showed no significant (time × group) interaction or main effects, indicative of no significant differences between mTBI and minor stroke over time. Overall, CLCE-24 cognition score showed a significant negative linear time effect and a positive quadratic time effect, indicative of a decrease in cognitive problems over time, which levels off over time (Fig. 3).

**Fig. 4:**
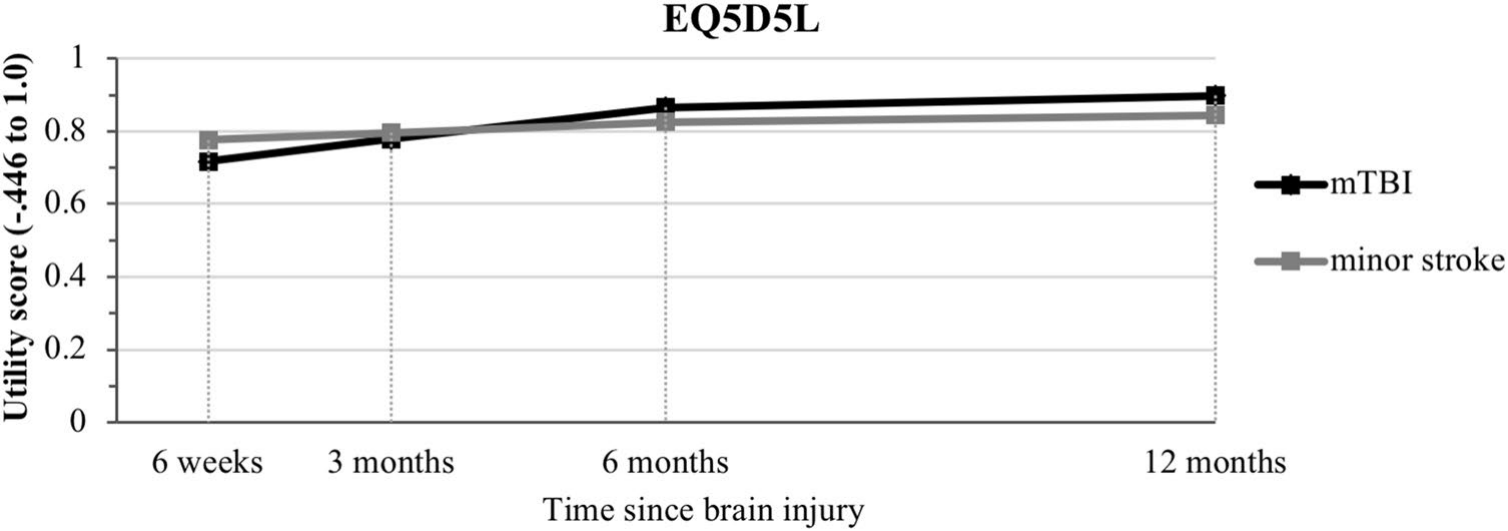
EuroQol 5 dimensions 5 levels (EQ-5D-5L) utility score displayed over time through multilevel growth curve model estimates

EQ-5D-5L showed a significant negative interaction term of the linear time effect with group, indicative of significantly less improvement over time in minor stroke compared to mTBI. The significant positive interaction term of quadratic time effect with group is indicative of stable levels in minor stroke minor stroke remaining on stable levels, whereas effects in mTBI leveled off over time on the EQ-5D-5L (Fig. 4).

No significant differences were observed between mTBI and minor stroke in proportions of persons reporting persistent symptoms at T2 on the HADS anxiety (*χ*^2^ (1) = 0.15, *p* = 0.70), HADS depression (*χ*^2^ (1) = 0.33, *p* = 0.57), CLCE-cognition (*χ*^2^ (1) = 3.61, *p* = 0.06), and EQ-5D-5L (*χ*^2^ (1) = 2.23, *p* = 0.14). Persistent symptoms on the HADS anxiety were reported in 17% of mTBI compared to 14% of minor stroke and on HADS depression by 15 and 12%, respectively. Persistent symptoms on the CLCE-24 cognition were reported by 57% of mTBI and 73% of minor stroke. The top five of experienced cognitive problems were equal for mTBI and minor stroke, despite varying percentages: doing two things at once (42% in mTBI vs. 49% in minor stroke), attending to things (43 vs. 46%), keeping up (mental slowness) (46 vs. 59%), remembering new information (43 vs. 56%) and forgetfulness (46 vs 39%). Persistent symptoms on the EQ-5D-5L regarding QoL was experienced by 38% in mTBI and 51% in minor stroke.

## Discussion

This study provides a first direct comparison of the long-term psychosocial outcome between mTBI and minor stroke. Psychosocial functioning generally improved up to 6 months, after which stabilization took place up to 12 months post-injury in both groups. On average, the mTBI and minor stroke cases seemed to recover well over time, especially for depressive symptoms and QoL. On average, significantly more severe anxiety symptoms and less improvement in QoL was observed over time in minor stroke compared to mTBI. No differences over time were observed between groups in severity of depressive symptoms and number of cognitive problems. Proportions of persons experiencing persistent psychosocial symptoms were similar between mTBI and minor stroke.

Compared with the general population aged 57–65 years, levels of anxiety symptoms were higher in both mTBI and minor strokes and remained higher up to 12 months post-injury [29]. Also, significant percentages of persons with minor stroke reported persistent anxiety symptoms, which could presumably be due a disproportional fear of stroke recurrence. Fear of stroke recurrence is an illness cognition, most commonly provoking anxiety in minor stroke, and which could lead to maladaptive avoidant behavior and phobic anxiety [30]. Illness cognitions are of interest in the mTBI population as well. Cognitions concerning the impact and duration of symptoms were shown to be highly predictive of persistent psychosocial symptoms following mTBI [31].

The number of cognitive problems in daily life was shown to be above general population levels, which is consistent with previous findings [28]. More than half of the samples experienced persistent cognitive problems in daily life despite the mild severity of the injuries. Moreover, mTBI and stroke experienced problems in similar cognitive domains: attention and memory. Persistent symptoms in daily life can impact QoL, as observed in significant percentages reporting problems regarding the QoL domains at 6 months post-injury, and which continue impacting QoL up to 5 years post-injury [15].

### Clinical implications

The observed similarities in progression of psychosocial outcome outweigh the differences, justifying a transdiagnostic design of uniform aftercare for mild brain injury, providing continued attention to long-term psychosocial outcome and persisting symptomatology in both mTBI and minor strokes. It is critical to actively monitor psychosocial functioning because psychosocial symptoms are not easily recognized and known to impact daily functioning and QoL [6, 32, 33]. Current stroke healthcare programs primarily focus on secondary prevention and functional outcome [34] and usually do not focus on long-term psychosocial symptoms. In mTBI, coordinated systems of care are deemed crucial, but deficiencies exist in the continuum of care and are suboptimal in reality [2]. Appropriate timing for providing uniform aftercare is considered to be at 6 months post-injury. At 6 months, functioning typically stabilizes and persons start encountering problems in daily life [6] which potentially persist into the long term if left unattended [6, 10, 11]. Uniform aftercare could be incorporated in primary health care which has been put forward by the WHO to ensure availability of quality and effective health care while containing healthcare costs [35]. The WHO also describes the essence of adopting a holistic and person-centered approach in supporting well-being [35]. Besides, support from specialized health care is only needed for the minority, according to the stepped-care model, while the majority is in need of low-intensity interventions [36] which fit the primary healthcare setting.

Primary care-based uniform aftercare can include low-intensity interventions such as monitoring, counseling through motivational interviewing, and psychoeducation, which have been shown to have beneficial psychosocial effects [37, 38]. It is essential to ensure specialists knowledge of brain injury in uniform aftercare. Despite significant proportions receiving psychological consultation, cognitive symptoms and anxiety remained above levels observed in the general population. Moreover, practitioners of uniform aftercare should be aware of, and pay specific attention to individual and brain injury-specific differences related to the underlying causes. For instance, the necessary attention for secondary prevention in the stroke population should be given in any case. Importantly, the heightened levels of anxiety in the stroke population should receive specific attention from the psychosocial perspective. Exposure techniques could be introduced through promotion of resuming activities of daily living [30]. Exposure is known to decrease anxiety [39] and promote self-management [40]. Psychoeducation could help persons in dealing with cognitive problems in daily life. Moreover, improving emotional well-being has also been associated with reduced cognitive symptoms experienced in daily life [41].

Accurate prognostic models could assist clinical management by identifying persons most in need of uniform aftercare. Prognostic models should take into account the complex nature of persistent psychosocial symptoms and look beyond hospital variables such as injury-related information, frequently shown to be unrelated to psychosocial outcome [31]. Instead, models should consider the biopsychosocial model and incorporate for example, illness perceptions.

### Strengths and limitations

This study concerned a multicenter, prospective, longitudinal cohort following persons from the ED up to 1-year post-injury. With multilevel growth modeling, we attempted to make best use of the available data and provide the most comprehensive overview of outcomes over time. Validated self-report measures of psychosocial functioning were used. It must be noted that the CLCE-24 is not validated in the TBI population but was selected to enable the comparison of common cognitive and emotional symptoms across conditions. Furthermore, the sample size of minor stroke, which was rather small compared to the mTBI group, potentially reduces statistical power. Further, our study was hospital based which may lead to missing large numbers of mTBI cases who do not visit the hospital. Ideally, population-based cohort studies are performed. Finally, in order to strengthen the direct comparison and in line, recommendations for uniform aftercare, future studies should extend covariates to, for example, premorbid psychological conditions and residual neurological deficits across conditions.

## Conclusion

This study showed that psychosocial outcome is largely comparable following mTBI and minor stroke. Uniform aftercare for mild brain injury seems appropriate, and when implemented, specific attention should be paid to anxiety symptoms and cognitive problems in daily life. Accurate prognostic models could help in identifying persons most in need of uniform aftercare and keep numbers of persons visiting feasible by offering stepped-care models.

## Data Availability

The data that support the findings of this study are available from the corresponding author upon reasonable request.
