# Correction to: Long-term psychosocial outcome following mild traumatic brain injury and minor stroke: a direct longitudinal comparison

**DOI:** 10.1007/s00415-021-10741-0

**Published:** 2021-09-01

**Authors:** Daan P. J. Verberne, Rudolf W. H. M. Ponds, Mariëlle E. A. L. Kroese, Melloney L. M. Wijenberg, Dennis G. Barten, Raphaël Pasmans, Julie Staals, Caroline M. van Heugten

**Affiliations:** 1 Faculty of Health, Medicine and Life Sciences, School for Mental Health and Neuroscience (MHeNs), Maastricht University Medical Center, Maastricht University, P.O. 616 UNS 40, 6200 MD Maastricht, The Netherlands; 2 Limburg Center for Brain Injury, Maastricht, The Netherlands; 3 Department of Brain Injury Rehabilitation, Adelante Rehabilitation Centre of Expertise in Rehabilitation and Audiology, Hoensbroek, The Netherlands; 4 Department of Medical Psychology, Maastricht University Medical Centre, Maastricht, The Netherlands; 5 Department of Health Services Research, Faculty of Health, Medicine and Life Sciences, Maastricht University, Maastricht, The Netherlands; 6 Department of Neuropsychology and Psychopharmacology, Faculty of Psychology and Neuroscience, Maastricht University, Maastricht, The Netherlands; 7 Department of Emergency Medicine, VieCuri Medical Center, Venlo, The Netherlands; 8 Department of Neurology, Zuyderland Medical Center, Heerlen, The Netherlands; 9 Department of Neurology, Maastricht University Medical Center, Maastricht, The Netherlands

## Correction to: Journal of Neurology 10.1007/s00415-020-10385-6

The original version of this article unfortunately contained a mistake in Table 2. The corrected Table 2 is given in the following page.

**Table 2:** Multilevel growth modeling of outcomes over time and interaction effects with injury type

	Time effects						Group effect			Interaction effects					
	Linear			Quadratic			Main (stroke = 1)			Linear × group			Quadratic × group		
	Estimate	SE	95% CI	Estimate	SE	95% CI	Estimate	SE	95% CI	Estimate	SE	95% CI	Estimate	SE	95% CI
HADS anxiety	− 0.31	0.11	− **0.52 to **− **0.11**	0.02	0.01	**0.01 to 0.04**	1.70	0.77	**0.18 to 3.21**	− 0.20	0.23	− 0.64 to 0.25	0.01	0.02	− 0.02 to 0.04
HADS depression	− 0.37	0.11	− **0.59 to **− **0.15**	0.03	0.01	**0.01 to 0.04**	0.10	0.81	− 1.51 to 1.70	0.20	0.24	− 0.28 to 0.68	− 0.02	0.02	− 0.05 to 0.02
CLCE-24 cognition	− 0.30	0.10	− **0.49 to **− **0.10**	0.02	0.01	**0.01 to 0.03**	0.12	0.69	− 1.24 to 1.48	0.20	0.21	− 0.22 to 0.62	− 0.01	0.02	− 0.04 to 0.01
EQ5D5L	0.04	0.01	**0.03 to 0.06**	− 0.003	0.0004	− **0.003 to **− **0.002**	0.06	0.04	− 0.01 to 0.13	− 0.03	0.01	− **0.05 to **− **0.007**	0.002	0.001	**0.0002 to 0.003**

Model covariates: age, sex, level of education, relationship status and neurological history. Significant estimates are displayed by 95% CI in bold (zero not included)

*CI* confidence interval, *CLCE-24* Checklist for Cognitive and Emotional Consequences of Stroke, *EQ-5D-5L* EuroQol 5 domains 5 levels, *HADS* Hospital Anxiety and Depression Scale, *SE* standard error

